# Current Understanding of piRNA in Cardiovascular Diseases

**DOI:** 10.3389/fmmed.2021.791931

**Published:** 2022-01-12

**Authors:** Iokfai Cheang, Qingqing Zhu, Shengen Liao, Xinli Li

**Affiliations:** First Affiliated Hospital, Nanjing Medical University, Nanjing, China

**Keywords:** transcriptome, non-coding RNA, piRNA, cardiovascular disease, Piwi associated RNAs

## Abstract

The relationship regarding non-coding genomes and cardiovascular disease (CVD) has been explored in the past decade. As one of the leading causes of death, there remains a lack of sensitive and specific genomic biomarkers in the diagnosis and prognosis of CVD. Piwi-interacting RNA (piRNA) is a group of small non-coding RNA (ncRNA) which associated with Piwi proteins. There is an emerging strong body of evidence in support of a role for ncRNAs, including piRNAs, in pathogenesis and prognosis of CVD. This article reviews the current evidence for piRNA-regulated mechanisms in CVD, which could lead to the development of new therapeutic strategies for prevention and treatment.

## Transcriptomics and PIWI-Interacting RNAs

Transcriptomics refer to the total amount of RNA transcribed by a specific tissue or cell in a certain stage or functional state and includes protein-coding messenger RNAs (mRNAs) and non-coding RNAs (ncRNAs) (Suzuki and Sugano, 2006). The functional significance of non-coding RNAs is particularly evident for small regulatory RNAs.

With its rapid development, omics has launched the molecular understanding of disease phenotypes into a new era. While the analysis of RNA Atlas data have expanded the catalog and their roles in regulating protein-coding genes and pathways (Lorenzi et al., 2021), integration of transcriptome profiling enabled analyses toward ncRNA for functional evidence is still being explored. PIWI protein-interacting RNAs (piRNAs) have been identified as an important class of small regulatory RNAs, together with microRNAs (miRNAs) and short-interfering RNAs (siRNAs).

piRNAs are an emerging class of small non-coding RNAs that started to get noticed in 2006. Most piRNAs consist with 24–32 nucleotide in length and represent a distinct small‐RNA pathway. In mammals, piRNAs mainly express in germline tissue such as human adult testis and fetal ovary. In addition, several studies have confirmed that piRNAs are also expressed in the brain, liver, heart, and vascular tissues (Ghosheh et al., 2016; Rajan et al., 2016; Kim, 2019; Lipps et al., 2019; Li et al., 2021). Non-repetitive piRNA genes account for over 90% of piRNAs (Ozata et al., 2019). The human genome encodes four PIWI proteins, PIWIL1/HIWI, PIWIL2/HILI, PIWIL3/HIWI3, and PIWIL4/HIWI2 (Sasaki et al., 2003; Grimson et al., 2008), which corresponds piRNA to form piRNA/PIWI complexes that are associated with transposon silencing, spermatogenesis, genome rearrangement, epigenetic regulation, mRNA regulation and development, and types of genomic sequences integrity.

### Functions of piRNAs

Historically, non-coding regions of the human genome, including those encoding piRNAs, were considered junk DNA. However, the development of high-throughput technologies over the past decades provided us an initial understanding of noncoding genomes. The crucial roles of the piRNA/PIWI pathway reflected in mediating the regulation of mRNA, lncRNA, and satellite RNA homeostasis by transposons and pseudogenes via transcriptional or posttranscriptional mechanisms.

piRNAs also play the protective role in germline genome integrity and stability by transposon silencing and epigenetic regulation (Qian et al., 2021; Rosenkranz et al., 2021). Cytoplasmic piRNA/PIWI complex can fulfill its function in transposon silencing through multiple pathways including: Hsp90-HOP to influence canalization; interact with translational initiators to induce inhibit polysomes and subsequent protein translation; piRNAs sequence-specific silencing to maintain genomic integrity and produce antiviral immune memories; piRNA-induced silence compounds via mitochondria to suppress transposons.

On the other hand, the role for piRNA repressing transposons in the nucleus seems to be more comprehensive. Regulation of heterochromatin protein-1 (HP), H3K9 methylation, ZF-MIWI2-mediated DNA methylation, Ccr4-NOT deadenylase complexes in multi-pathway (the active full-length transposable element, telomere, and sub telomere accumulation), UHRF1/PRMT5/PIWI-mediated histone methylation, etc., are all considered to influence transcription of selected protein-encoding genes, imprinting loci, and/or transposons (Iwasaki et al., 2016; Kojima-Kita et al., 2016; Penke et al., 2016; Dong et al., 2019). Furthermore, these studies in the field of ncRNA verified that piRNA plays an indispensable role in germ cell and stem cell differentiation, embryonic development, germline DNA integrity, biological sex determination, immune defense, and cancer progression (Batki et al., 2019).

### Cardiovascular Disease and Transcriptome

Cardiovascular disease (CVD) remains the leading cause of death worldwide. Over 17 million people die of cardiovascular disease worldwide each year, similar to the death rate from all cancers combined (Batki et al., 2019). In China, due to population aging and changes in dietary structure, the morbidity and mortality of CVDs including hypertension, coronary heart disease, and congestive heart failure have shown upward trends (Hu et al., 2020), which imposes additional social and financial burdens. The prevention and treatment of CVDs is still a major task of modern medicine.

Emerging novel transcriptomics research on CVD have shown its unique diagnostic and prognostic value (Schnabel et al., 2012; Gomes et al., 2019; Wiese et al., 2019). Studies regarding the diagnostic values of miRNA or long non-coding RNA (lncRNA) in CVD have published in GEO (Gene Expression Omnibus) database. Furthermore, study suggested that the epitope transcriptome analysis could be performed on admission to provide information of RNA modification in individual transcriptome (Gatsiou and Stellos, 2018). Exploring the multifaceted functions of these RNAs in the pathogenesis of CVD may fulfill a promising clinical applications as diagnostic biomarkers and therapeutic targets.

### Evidence of piRNA in CVD

Cardiac hypertrophy and fibrosis are the fundamental pathophysiological adaptive responses during the development of various CVDs, piRNA have shown to participate in such process suggesting that piRNAs play an important role in the disease occurrence (Rai et al., 2017; Kwiecinski et al., 2020; Zeng et al., 2021). Study has demonstrated that a group of piRNAs expression was altered during induced cardiac hypertrophy. The downregulation of hsa-piR-020009 and hsa-piR-006426 was also potentially involved in heart failure (Yang et al., 2018). piRNA-2106027 was involved in the occurrence and development of myocardial infarction through troponin-1 (Rajan et al., 2016). In addition, there were 21 piRNAs found to be differentially expressed in chronic thromboembolic pulmonary hypertension (CTEPH) patients compared to the controls. piRNA-DQ593039-SNX17-SERCA pathway showed to be associated with the development of cardiac and vascular tissue remodeling (Lipps et al., 2019). These piRNAs have been recognized as significant players in gene regulation by pairing with the complementary base of the target RNA or binding to the target protein, which may profoundly impact the cellular responses and thus both health and disease course (Table 1).

**TABLE 1:** Description of piRNAs and CVD literature articles.

Category	Specific piRNA	Article type (description)	Diseases/Target	Author(s), year published, and PMID
Evidence of piRNA in CVD	• 585 piRNAs upregulated	cross-sectional study (serum of heart failure patients and healthy volunteers)	Heart Failure	Yang et al. (2018), 30536343
	• 4,623 piRNAs downregulated (has-piR-020009 and has-piR-006426 were the most downregulated)			
	• piRNA-2106027	cross-sectional study (circulation of 6 myocardial infarction patients and 5 controls)	Myocardial Infarction	Rajan et al. (2016), 27067666
	• Has-piR-33151 (DQ593039) highly upregulated (biomarker for lung and heart diseases)	cross-sectional study (extracellular vesicles form 23 patients with CTEPH and 23 controls)	Chronic thrombo-embolic pulmonary hypertension (CTEPH)	Lipps et al. (2019), 31671920
	• Has-piR-33543 (DQ593431) upregulated			
	• Has-piR-31068 (DQ570956) downregulated			
Mechanisms involved- piRNAs in CVD	• mmu-piR-037808 (CHAPIR)	Basic research (CHAPIR-METTL3-PARP10-NFATC4 signal axis)	Cardiac Hypertrophy	Gao et al. (2020), 33020597
	• piRNA-823	Basic research and case-control study (bone marrow biopsy specimens from 15 newly diagnosed multiple myeloma [MM] patients and 8 control as well as primary CD138^+^ MM cells; Plasma cells from 43 newly diagnosed cases of MM and 18 controls; Human MM cell lines: RPMI8226, ARH-77 and U266)	Angiogenesis	Yan et al. (2015), 24732595
	• LINE-1 retrotransposon	Basic research (inhibition of LINE-1 retrotransposon)	Ischaemic Damage	Lucchinetti et al. (2006), 16418318
	• Akt pathway	Basic research (activation of the Akt/PKB signaling; inhibition of cell apoptosis and epigenetic modifications)	Ischaemic Damage	Rajan et al. (2014), 25220478; Zhu et al. (2006), 16901477

### Regulatory Mechanism of piRNAs in CVD

**FIGURE 1:**
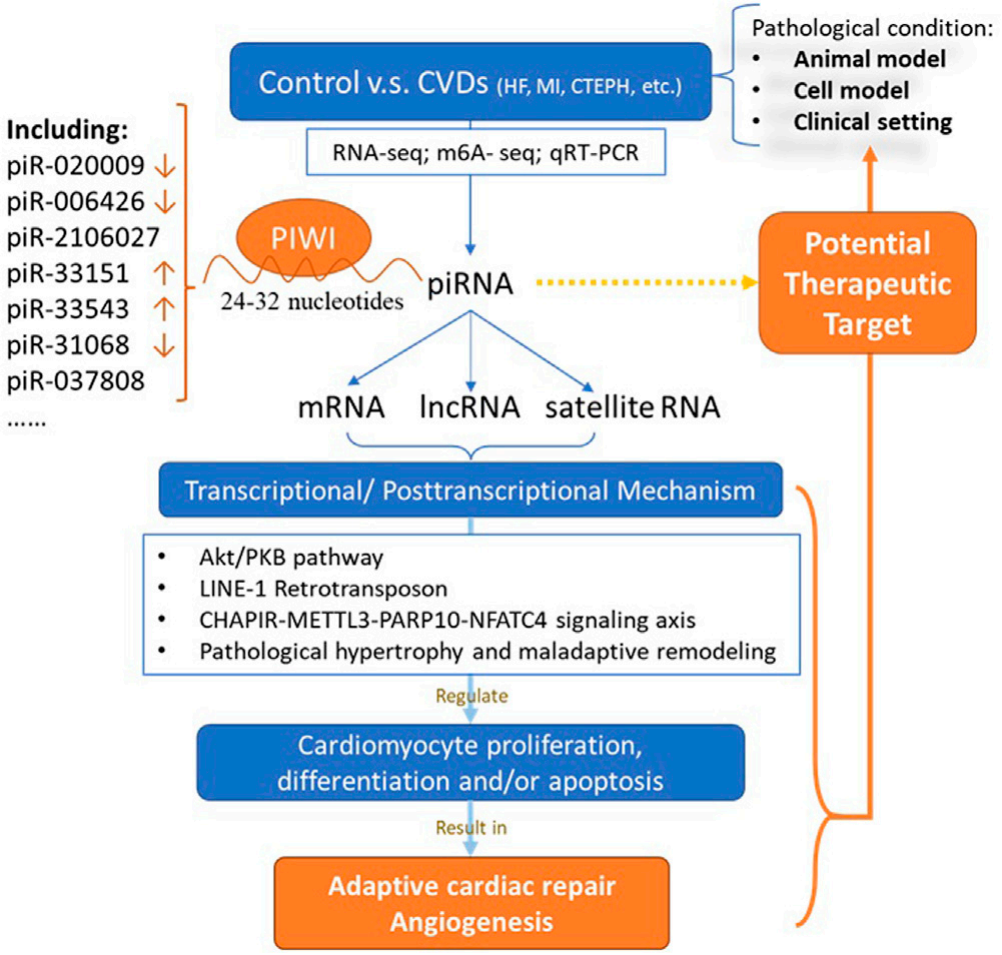
Role of piRNA in cardiovascular diseases. CVD: cardiovascular disease; piRNA: PIWI-interacting RNAs; mRNA: messenger RNA; lncRNA: long non-coding RNA.

Multiple regulatory functions of gene expression in diverse organisms were exhibited in piRNA/PIWI pathway (Figure 1). Genome histone modifications and DNA methylation have shown to be the major regulating mechanism (Juliano et al., 2011; Li et al., 2021). Growing evidence also demonstrated that piRNAs involve in regulating cell proliferation, migration, apoptosis, cell cycle, oxidative stress, and DNA damage (Wu et al., 2020). Gao XQ et al. found that the RNA epigenetic mechanism mediated by mmu-piR-037808 (CHAPIR) is involved in the regulation of cardiac hypertrophy (Gao et al., 2020). They constructed CHAPIR knockout (CHAPIR KO) mice with reduced cardiac fibrosis and hypertrophy after transverse aortic constriction (TAC) surgery. At the cellular level, the similar function was also found in Ang-II-induced hypertrophic growth of cardiomyocytes. The CHAPIR - METTL3-PARP10-NFATC4 signal axis may be considered as therapeutic target used to treat pathological hypertrophy and maladaptive cardiac remodeling in the future.

Study also showed that abrogation of piRNA-823 can induce the reduction of vascular endothelial growth factor secretion, thus reducing the pro-angiogenesis activity in multiple myeloma (MM) cells (Yan et al., 2015). In addition, studies have shown that methyl-CpG binding protein 2 (MeCP2) expressed in the heart is an inhibitor of long interspersed element-1 (LINE-1, the only autonomous reverse transposon in the human genome). LINE-1 was reported to reduce ischemic injury by activating Akt/PKB signaling (Lucchinetti et al., 2006). In the absence of MeCP2, the Piwi-piRNA pathway may be activated to initiate Akt signaling in the cardiac system (Zhao et al., 2013; Rajan et al., 2014). Similarly, piRNA targets transposons——LINE-1 in cardiac progenitor cells. Reduced LINE-1 expression is accompanied by higher activation of the Akt pathway (Vella et al., 2016). Therefore, piRNAs may regulate myocardial ischemia in the Akt pathway via inhibiting cell apoptosis and epigenetic modifications (Zhu et al., 2006; Rajan et al., 2014). Overall, these mechanisms may shed new light on the understanding regulatory mechanism of piRNAs in CVD.

### Study Pattern of piRNAs in CVD

While most understandings regarding piRNA in humans were as a predictive molecule for diagnosis and prognosis of neoplastic diseases, current studies of piRNAs on CVD are only at animal and cellular levels. Therefore, there is still room for improvement in the development of piRNA clinical research on CVDs. By merging the application experience in tumors, it might further verify the expression of key piRNAs and their target genes in CVD related pathways and biological processes. At present, the main research methods for the effect of piRNAs on CVD include small RNA sequencing, validation by quantitative real-time polymerase chain reaction (qRT-PCR), target gene prediction, target gene functional analysis, and correlation analysis with mRNA. Together, further exploration using single-cell resolution to study expression levels, such as *in situ* hybridization or single-cell RNA sequencing, can help to clarify the presence or absence of related piRNAs expression. Transcriptome-wide m6A-seq and RNA-seq assays are also used to identify potential targets of piRNA (Ghosheh et al., 2016). Besides, the function of these piRNAs can also be mapped by bioinformatics network and pathway analysis. Predictive algorithms including Miranda and RNA22 program can be applied to search for targets of differential expression (DE) piRNA against all human transcripts (Alexandrova et al., 2016; Meng et al., 2019; Das et al., 2020). Strict thermodynamic parameters and binding energy thresholds can be applied to predict potential targets that are complementary to piRNAs.

### Potential of piRNA in Cardiovascular Therapy

Traditionally, the adult heart was considered with no capacity for cardiomyogenesis to compensate for cell lost in cardiac diseases. However, novel animal and human studies with lineage tracing and isotope labeling methods supported the heart has certain endogenous regenerative potential (Laflamme and Murry, 2011; Rosenzweig, 2012). Unfortunately, this regeneration was temporary observed in the early postnatal mammalian heart. The proliferation capacity of the adult mammalian heart is very limited to achieve effective self-repair of the myocardium after injury and resist the development of heart failure (Drenckhahn et al., 2008; Vandergriff et al., 2018). To be noted, the eutrophic environment from an anaerobic environment is a curial factor that causes the inhibition of cardiomyocytes proliferation (Puente et al., 2014; Paradis et al., 2015). At the molecular level, cytokines and transcription factors (e.g., FGF2, PDGF, NRG1, Meis1) related to growth and differentiation are important regulatory factors for the proliferation of neonatal mammalian cardiomyocytes, while piRNAs were also shown to involve in (Kardami et al., 2003; Srisakuldee et al., 2006; Hinrichsen et al., 2007; Odiete et al., 2012; Ghosheh et al., 2016).

As a multi-mechanism regulatory element, piRNAs not only participated in tumorigenesis but also regulate cardiac differentiation in certain cardiomyogenesis settings including ischemic damage and heart dysfunction remodeling. Such as piR-1078 was discovered to be differentially expressed in the process of cardiac development (Pipicz et al., 2018; Zeng et al., 2021). The corresponding PIWI protein also play certain role in the regulation of non-repetitive transcription in stem cells, such as the expression level of PIWIL2 might participle in this transposon silencing in iPS cells (Marchetto et al., 2013; Gomes Fernandes et al., 2018).

Promoting the adequate proliferation of cardiomyocytes may be a new treatment target to prevent abnormal hypertrophy and pathological fibrosis. To determine the molecular basis of pluripotent stem cells, it could provide an important understanding of the characteristics in cardiovascular system at the RNA level and connect the existing evidence of reference genes. Further research along these directions regarding the function of stem cells and piRNA/PIWI proteins pathway can supplement our knowledge in cardiovascular therapy.

## Conclusion

There is much to be discovered about the existence and function of PIWI protein in somatic cells. First, variety of pathological heart conditions and the related mechanisms have not been elucidated, and the roles of these piRNA/PIWI proteins therein are not conclusive yet. Conjoint assays of in-depth small RNA sequencing and m6A-seq and other technologies, piRNAs and the corresponding mechanism of transposon silencing and epigenetic regulation could be unveiled. Simultaneously, the exploration of piRNAs involved in stem cell-derived cardiomyocytes is of great promoting value to the molecular mechanisms underlying cardiac repair.

Integrating research focused on these areas will provide the potential applications of piRNAs in the clinical diagnosis, prognosis, and therapeutic strategies for cardiovascular diseases.

## References

[B1] Alexandrova E. Miglino N. Hashim A. Nassa G. Stellato C. Tamm M. (2016). Small RNA Profiling Reveals Deregulated Phosphatase and Tensin Homolog (PTEN)/Phosphoinositide 3-Kinase (PI3K)/Akt Pathway in Bronchial Smooth Muscle Cells from Asthmatic Patients. J. Allergy Clin. Immunol. 137 (1), 58–67. 10.1016/j.jaci.2015.05.031 26148798

[B3] Batki J. Schnabl J. Wang J. Handler D. Andreev V. I. Stieger C. E. (2019). The Nascent RNA Binding Complex SFiNX Licenses piRNA-Guided Heterochromatin Formation. Nat. Struct. Mol. Biol. 26 (8), 720–731. 10.1038/s41594-019-0270-6 31384064 PMC6828549

[B4] Das B. Jain N. Mallick B. (2020). piR‐39980 Promotes Cell Proliferation, Migration and Invasion, and Inhibits Apoptosis via Repression of SERPINB1 in Human Osteosarcoma. Biol. Cel 112 (3), 73–91. 10.1111/boc.201900063 31879982

[B5] Dong J. Wang X. Cao C. Wen Y. Sakashita A. Chen S. (2019). UHRF1 Suppresses Retrotransposons and Cooperates with PRMT5 and PIWI Proteins in Male Germ Cells. Nat. Commun. 10 (1), 4705. 10.1038/s41467-019-12455-4 31624244 PMC6797737

[B6] Drenckhahn J.-D. Schwarz Q. P. Gray S. Laskowski A. Kiriazis H. Ming Z. (2008). Compensatory Growth of Healthy Cardiac Cells in the Presence of Diseased Cells Restores Tissue Homeostasis during Heart Development. Develop. Cel 15 (4), 521–533. 10.1016/j.devcel.2008.09.005 18854137

[B7] Gao X.-Q. Zhang Y.-H. Liu F. Ponnusamy M. Zhao X.-M. Zhou L.-Y. (2020). The piRNA CHAPIR Regulates Cardiac Hypertrophy by Controlling METTL3-dependent N6-Methyladenosine Methylation of Parp10 mRNA. Nat. Cel Biol 22 (11), 1319–1331. 10.1038/s41556-020-0576-y 33020597

[B8] Gatsiou A. Stellos K. (2018). Dawn of Epitranscriptomic Medicine. Circ. Genom Precis Med. 11 (9), e001927. 10.1161/CIRCGEN.118.001927 30354331

[B9] Ghosheh Y. Seridi L. Ryu T. Takahashi H. Orlando V. Carninci P. (2016). Characterization of piRNAs Across Postnatal Development in Mouse Brain. Sci. Rep. 6, 25039. 10.1038/srep25039 27112104 PMC4844963

[B10] Gomes C. P. C. Ágg B. Andova A. Arslan S. Baker A. Barteková M. (2019). Catalyzing Transcriptomics Research in Cardiovascular Disease: The CardioRNA COST Action CA17129. Noncoding RNA 5 (2), 31. 10.3390/ncrna5020031 30934986 PMC6630366

[B11] Gomes Fernandes M. He N. Wang F. Van Iperen L. Eguizabal C. Matorras R. (2018). Human-Specific Subcellular Compartmentalization of P-Element Induced Wimpy Testis-Like (PIWIL) Granules during Germ Cell Development and Spermatogenesis. Hum. Reprod. 33 (2), 258–269. 10.1093/humrep/dex365 29237021 PMC5850288

[B12] Grimson A. Srivastava M. Fahey B. Woodcroft B. J. Chiang H. R. King N. (2008). Early Origins and Evolution of microRNAs and Piwi-Interacting RNAs in Animals. Nature 455 (7217), 1193–1197. 10.1038/nature07415 18830242 PMC3837422

[B13] Hinrichsen R. HaunsØ S. Hinrichsen R. Hinrichsen R. HaunsØ S. Busk P. K. (2007). Different Regulation of P27 and Akt during Cardiomyocyte Proliferation and Hypertrophy. Growth Factors 25 (2), 132–140. 10.1080/08977190701549835 17852410

[B2] Hu S. Chen Y. Gao R. Ge J. Gu D. Han Y. (2020). The Writing Committee of the Report on Cardiovascular Health and Diseases in China. Report on Cardiovascular Health and Diseases in China 2019: an Updated Summary[J]. Chin. Circ. J. 35 (9), 833–854. 10.3969/j.issn.1000-3614.2020.09.001

[B14] Iwasaki Y. W. Murano K. Ishizu H. Shibuya A. Iyoda Y. Siomi M. C. (2016). Piwi Modulates Chromatin Accessibility by Regulating Multiple Factors Including Histone H1 to Repress Transposons. Mol. Cel 63 (3), 408–419. 10.1016/j.molcel.2016.06.008 27425411

[B15] Juliano C. Wang J. Lin H. (2011). Uniting Germline and Stem Cells: The Function of Piwi Proteins and the piRNA Pathway in Diverse Organisms. Annu. Rev. Genet. 45, 447–469. 10.1146/annurev-genet-110410-132541 21942366 PMC3832951

[B16] Kardami E. Banerji S. Doble B. W. Dang X. Fandrich R. R. Jin Y. (2003). PKC-Dependent Phosphorylation May Regulate the Ability of Connexin43 to Inhibit DNA Synthesis. Cel Commun. Adhes. 10 (4-6), 293–297. 10.1080/cac.10.4-6.293.297 14681031

[B17] Kim K. W. (2019). PIWI Proteins and piRNAs in the Nervous System. Mol. Cell 42 (12), 828–835. 10.14348/molcells.2019.0241 PMC6939654 31838836

[B18] Kojima-Kita K. Kuramochi-Miyagawa S. Nagamori I. Ogonuki N. Ogura A. Hasuwa H. (2016). MIWI2 as an Effector of DNA Methylation and Gene Silencing in Embryonic Male Germ Cells. Cel Rep. 16 (11), 2819–2828. 10.1016/j.celrep.2016.08.027 27626653

[B19] Kwiecinski J. Lennen R. J. Gray G. A. Borthwick G. Boswell L. Baker A. H. (2020). Progression and Regression of Left Ventricular Hypertrophy and Myocardial Fibrosis in a Mouse Model of Hypertension and Concomitant Cardiomyopathy. J. Cardiovasc. Magn. Reson. 22 (1), 57. 10.1186/s12968-020-00655-7 32758255 PMC7409657

[B20] Laflamme M. A. Murry C. E. (2011). Heart Regeneration. Nature 473 (7347), 326–335. 10.1038/nature10147 21593865 PMC4091722

[B21] Li M. Yang Y. Wang Z. Zong T. Fu X. Aung L. H. H. (2021). Piwi-interacting RNAs (piRNAs) as Potential Biomarkers and Therapeutic Targets for Cardiovascular Diseases. Angiogenesis 24 (1), 19–34. 10.1007/s10456-020-09750-w 33011960

[B22] Lipps C. Northe P. Figueiredo R. Rohde M. Brahmer A. Krämer-Albers E. M. (2019). Non-Invasive Approach for Evaluation of Pulmonary Hypertension Using Extracellular Vesicle-Associated Small Non-Coding RNA. Biomolecules 9 (11), 666. 10.3390/biom9110666 31671920 PMC6920761

[B23] Lorenzi L. Chiu H.-S. Avila Cobos F. Gross S. Volders P.-J. Cannoodt R. (2021). The RNA Atlas Expands the Catalog of Human Non-Coding RNAs. Nat. Biotechnol. 39, 1453–1465. 10.1038/s41587-021-00936-1 34140680

[B24] Lucchinetti E. Feng J. Silva R. d. Tolstonog G. V. Schaub M. C. Schumann G. G. (2006). Inhibition of LINE-1 Expression in the Heart Decreases Ischemic Damage by Activation of Akt/PKB Signaling. Physiol. Genomics 25 (2), 314–324. 10.1152/physiolgenomics.00251.2005 16418318

[B25] Marchetto M. C. N. Narvaiza I. Denli A. M. Benner C. Lazzarini T. A. Nathanson J. L. (2013). Differential L1 Regulation in Pluripotent Stem Cells of Humans and Apes. Nature 503 (7477), 525–529. 10.1038/nature12686 24153179 PMC4064720

[B26] Meng X. Peng H. Ding Y. Zhang L. Yang J. Han X. (2019). A Transcriptomic Regulatory Network Among miRNAs, piRNAs, circRNAs, lncRNAs and mRNAs Regulates Microcystin-Leucine Arginine (MC-LR)-Induced Male Reproductive Toxicity. Sci. Total Environ. 667, 563–577. 10.1016/j.scitotenv.2019.02.393 30833255

[B27] Odiete O. Hill M. F. Sawyer D. B. (2012). Neuregulin in Cardiovascular Development and Disease. Circ. Res. 111 (10), 1376–1385. 10.1161/CIRCRESAHA.112.267286 23104879 PMC3752394

[B28] Ozata D. M. Gainetdinov I. Zoch A. O’Carroll D. Zamore P. D. (2019). PIWI-interacting RNAs: Small RNAs with Big Functions. Nat. Rev. Genet. 20 (2), 89–108. 10.1038/s41576-018-0073-3 30446728

[B29] Paradis A. N. Gay M. S. Wilson C. G. Zhang L. (2015). Newborn Hypoxia/Anoxia Inhibits Cardiomyocyte Proliferation and Decreases Cardiomyocyte Endowment in the Developing Heart: Role of Endothelin-1. PLoS One 10 (2), e0116600. 10.1371/journal.pone.0116600 25692855 PMC4334650

[B30] Penke T. J. R. McKay D. J. Strahl B. D. Matera A. G. Duronio R. J. (2016). Direct Interrogation of the Role of H3K9 in Metazoan Heterochromatin Function. Genes Dev. 30 (16), 1866–1880. 10.1101/gad.286278.116 27566777 PMC5024684

[B31] Pipicz M. Demján V. Sárközy M. Csont T. (2018). Effects of Cardiovascular Risk Factors on Cardiac STAT3. Int. J. Mol. Sci. 19 (11), 3572. 10.3390/ijms19113572 30424579 PMC6274853

[B32] Puente B. N. Kimura W. Muralidhar S. A. Moon J. Amatruda J. F. Phelps K. L. (2014). The Oxygen-Rich Postnatal Environment Induces Cardiomyocyte Cell-Cycle Arrest through DNA Damage Response. Cell 157 (3), 565–579. 10.1016/j.cell.2014.03.032 24766806 PMC4104514

[B33] Qian L. Xie H. Zhang L. Zhao Q. Lü J. Yu Z. (2021). Piwi-Interacting RNAs: A New Class of Regulator in Human Breast Cancer. Front. Oncol. 11, 695077. 10.3389/fonc.2021.695077 34295823 PMC8290475

[B34] Rai V. Sharma P. Agrawal S. Agrawal D. K. (2017). Relevance of Mouse Models of Cardiac Fibrosis and Hypertrophy in Cardiac Research. Mol. Cel Biochem 424 (1-2), 123–145. 10.1007/s11010-016-2849-0 PMC5219849 27766529

[B35] Rajan K. S. Velmurugan G. Pandi G. Ramasamy S. (2014). miRNA and piRNA Mediated Akt Pathway in Heart: Antisense Expands to Survive. Int. J. Biochem. Cel Biol. 55, 153–156. 10.1016/j.biocel.2014.09.001 25220478

[B36] Rajan K. S. Velmurugan G. Gopal P. Ramprasath T. Babu D. D. V. Krithika S. (2016). Abundant and Altered Expression of PIWI-Interacting RNAs during Cardiac Hypertrophy. Heart Lung Circ. 25 (10), 1013–1020. 10.1016/j.hlc.2016.02.015 27067666

[B37] Rosenkranz D. Zischler H. Gebert D. (2021). piRNAclusterDB 2.0: Update and Expansion of the piRNA Cluster Database. Nucleic Acids Res., gkab622. 10.1093/nar/gkab622 PMC8728273 34302483

[B38] Rosenzweig A. (2012). Cardiac Regeneration. Science 338 (6114), 1549–1550. 10.1126/science.1228951 23258880

[B39] Sasaki T. Shiohama A. Minoshima S. Shimizu N. (2003). Identification of Eight Members of the Argonaute Family in the Human Genome☆. Genomics 82 (3), 323–330. 10.1016/s0888-7543(03)00129-0 12906857

[B40] Schnabel R. B. Baccarelli A. Lin H. Ellinor P. T. Benjamin E. J. (2012). Next Steps in Cardiovascular Disease Genomic Research-Sequencing, Epigenetics, and Transcriptomics. Clin. Chem. 58 (1), 113–126. 10.1373/clinchem.2011.170423 22100807 PMC3650722

[B41] Srisakuldee W. Nickel B. E. Fandrich R. R. Jiang Z. S. Kardami E. (2006). Administration of FGF-2 to the Heart Stimulates Connexin-43 Phosphorylation at Protein Kinase C Target Sites. Cell Commun Adhes 13 (1-2), 13–19. 10.1080/15419060600631326 16613776

[B42] Suzuki Y. Sugano S. (2006). Transcriptome Analyses of Human Genes and Applications for Proteome Analyses. Curr. Protein Pept. Sci. 7 (2), 147–163. 10.2174/138920306776359795 16611140

[B43] Vandergriff A. Huang K. Shen D. Hu S. Hensley M. T. Caranasos T. G. (2018). Targeting Regenerative Exosomes to Myocardial Infarction Using Cardiac Homing Peptide. Theranostics 8 (7), 1869–1878. 10.7150/thno.20524 29556361 PMC5858505

[B44] Vella S. Gallo A. Lo Nigro A. Galvagno D. Raffa G. M. Pilato M. (2016). PIWI-interacting RNA (piRNA) Signatures in Human Cardiac Progenitor Cells. Int. J. Biochem. Cel Biol. 76, 1–11. 10.1016/j.biocel.2016.04.012 27131603

[B45] Wiese C. B. Zhong J. Xu Z.-Q. Zhang Y. Ramirez Solano M. A. Zhu W. (2019). Dual Inhibition of Endothelial miR-92a-3p and miR-489-3p Reduces Renal Injury-Associated Atherosclerosis. Atherosclerosis 282, 121–131. 10.1016/j.atherosclerosis.2019.01.023 30731284 PMC7484899

[B46] Wu X. Pan Y. Fang Y. Zhang J. Xie M. Yang F. (2020). The Biogenesis and Functions of piRNAs in Human Diseases. Mol. Ther. - Nucleic Acids 21, 108–120. 10.1016/j.omtn.2020.05.023 32516734 PMC7283962

[B47] Yan H. Wu Q.-L. Sun C.-Y. Ai L.-S. Deng J. Zhang L. (2015). piRNA-823 Contributes to Tumorigenesis by Regulating De Novo DNA Methylation and Angiogenesis in Multiple Myeloma. Leukemia 29 (1), 196–206. 10.1038/leu.2014.135 24732595

[B48] Yang J. Xue F. T. Li Y. Y. Liu W. Zhang S. (2018). Exosomal piRNA Sequencing Reveals Differences between Heart Failure and Healthy Patients. Eur. Rev. Med. Pharmacol. Sci. 22 (22), 7952–7961. 10.26355/eurrev_201811_16423 30536343

[B49] Zeng Q. Cai J. Wan H. Zhao S. Tan Y. Zhang C. (2021). PIWI-Interacting RNAs and PIWI Proteins in Diabetes and Cardiovascular Disease: Molecular Pathogenesis and Role as Biomarkers. Clinica Chim. Acta 518, 33–37. 10.1016/j.cca.2021.03.011 33746016

[B50] Zhao L. Y. Zhang J. Guo B. Yang J. Han J. Zhao X. G. (2013). MECP2 Promotes Cell Proliferation by Activating ERK1/2 and Inhibiting P38 Activity in Human Hepatocellular Carcinoma HEPG2 Cells. Cel Mol Biol (Noisy-le-grand) 59, OL1876–81. 24199952

[B51] Zhu M. Feng J. Lucchinetti E. Fischer G. Xu L. Pedrazzini T. (2006). Ischemic Postconditioning Protects Remodeled Myocardium via the PI3K-PKB/Akt Reperfusion Injury Salvage Kinase Pathway. Cardiovasc. Res. 72 (1), 152–162. 10.1016/j.cardiores.2006.06.027 16901477

